# Potential of *saccharomyces cerevisiae* fermentation-derived postbiotic technology in mitigating multiple drug-resistant *Salmonella enterica* serovars in an *in vitro* broiler cecal model

**DOI:** 10.1371/journal.pone.0320977

**Published:** 2025-04-03

**Authors:** Elena G. Olson, Dana K. Dittoe, W. Evan Chaney, Andrea M. Binnebose, Steven C. Ricke

**Affiliations:** 1 Animal and Dairy Sciences Department, Meat Science and Animal Biologics Discovery Program, University of Wisconsin-Madison, Madison, Wisconsin, United States of America; 2 Department of Animal Science, University of Wyoming, Laramie, Wyoming, United States of America; 3 Cargill, Inc., Micronutrition and Health Solutions, Wayzata, Minnesota, United States of America; Tokat Gaziosmanpaşa University: Tokat Gaziosmanpasa Universitesi, TÜRKIYE

## Abstract

Diamond V Original XPC® is a *Saccharomyces cerevisiae* fermentation-derived postbiotic technology (SCFP) designed to interact synergistically with the animal to provide health benefits by enhancing immune function, supporting digestive integrity and absorption, and maintaining gastrointestinal (GIT) microbial balance in the host. The current study investigated the effects of 1.25% SCFP on multidrug-resistant (MDR) *Salmonella* serovars: *S*. Typhimurium (ATCC 14028), *S*. Enteritidis, *S*. Infantis, *S*. Heidelberg, *S*. Typhimurium DT104, and *S*. Reading, and shifts in cecal microbiota populations. Using an anaerobic *in vitro* poultry cecal model, cecal contents were inoculated with ~ 10^8^ colony forming units (CFU) of MDR *Salmonella* serovars and incubated for 24 h at 37°C anaerobically. The treatments included: control group consisting of 0.2 g of crushed poultry feed, and a treatment group 0.25 g of feed +  1.25% inclusion of Original XPC® (SCFP). The SCFP significantly reduced five of the six serovars: *S*. Typhimurium ATCC, *S*. Enteritidis, *S*. Infantis, *S*. Heidelberg, and S. Reading (P <  0.05). Time significantly impacted *S*. Typhimurium DT104 reduction (P <  0.001). The most significant decrease was observed for *S*. Enteritidis (3.9 log_10_ CFU/mL), followed by *S*. Heidelberg (3.8 log_10_ CFU/mL), *S*. Infantis (3.4 log_10_ CFU/mL), *S*. Typhimurium ATCC (3 log_10_ CFU/mL), and *S*. Reading (1.8 log_10_ CFU/mL) compared to controls that averaged approximately 1 log_10_ CFU/mL reduction. Microbiota analysis at 24 h involved genomic DNA extraction, amplification using custom dual-indexed primers, and sequencing on the Illumina MiSeq platform. Sequencing data were analyzed using QIIME2-2021.11. *S*. Infantis and *S*. Heidelberg inoculated samples were the only groups that significantly enhanced microbial richness and evenness with SCFP addition at 24 h (P <  0.05). Pairwise comparisons revealed that samples inoculated with *S*. Reading and *S*. Typhimurium DT104 exhibited a minor change in microbial composition with SCFP, compared to other serovars that demonstrated increased microbial diversity with SCFP. Additionally, *S*. Infantis and *S*. Heidelberg inoculated samples exhibited phylogenetic diversity and microbial abundance with SCFP compared to controls at 24 h (P <  0.05). *Lachnospiraceae* CHKCI001 was significantly more abundant in SCFP-treated samples compared to controls (ANCOM, P <  0.05), suggesting SCFP impact on cecal fermenters and production of fermentation end products that may impact the ecosystem and inhibit pathogen growth. Although various serovars may exhibit somewhat different responses, SCFP effectively mitigated multiple MDR serovars of *Salmonella* under *in vitro* incubation conditions.

## Introduction

*Salmonella enterica* consists of numerous serovars associated with food animals such as poultry. The genomes of *Salmonella* serovars vary in size, ranging from 4,460 to 4,857 base pairs, leading to differences in their metabolic functionality, virulence, and pathogenesis characteristics [1]. These differences may further be associated with *Salmonella’s* specific niche in poultry. Several *Salmonella* serovars have been mainly linked to poultry production, including *S*. Typhimurium, *S*. Enteritidis, *S*. Infantis, *S*. Heidelberg, and *S*. Reading [2]. Over 91% of *Salmonella enterica* isolates associated with poultry have shown resistance to at least one antibiotic [3]. Although the abundance of antibiotic resistance can vary between commercial and organic chickens and across different production systems and geographical locations, *Salmonella* isolates may exhibit resistance to similar antibiotics [3–5]. Furthermore, Punchihewage-Don et al. (2024) observed that isolates from commercial and organic chickens exhibited resistance to ceftriaxone and trimethoprim/sulfamethoxazole, antibiotics used in humans to treat salmonellosis [3,6]. Although there is no substantial evidence that exposure to multidrug-resistant (MDR) *Salmonella* necessarily causes untreatable infections in humans, the virulence potential of specific serovars has become an increasing focus for government regulatory agencies, such as the United States Department of Agriculture (USDA) Food Safety and Inspection Service (FSIS). Recently, the FSIS declared specific *Salmonella* serotypes at certain concentrations as adulterants in raw poultry products [7]. Therefore, reducing the prevalence of MDR *Salmonella* serovars entering the post-harvest food chain is crucial.

Preharvest strategies are increasingly being emphasized to reduce foodborne *Salmonella* in poultry before they enter processing facilities [8]. Dietary interventions, including feed additives, generally target optimization of performance through immune and GIT microbiome modulatory mechanisms that may also minimize the colonization potential of various enteric pathogens, including *Salmonella* [9–11]. A standard postbiotic technology is a *Saccharomyces cerevisiae* fermentation-derived product, which contains inanimate cells, cell fragments, and metabolites from the fermentation of *S. cerevisiae* in combination with the media used in the fermentation (SCFP) (Original XPC® Diamond V, Cedar Rapids, IA, USA). Numerous studies have reported significant reductions in *Salmonella* prevalence in poultry when SCFP was included in the dietary ration [9,12–19]. Reductions have also been observed in *in vitro* models [12,20–23].

Utilizing an *in vitro* poultry cecal model similar to ones used in previous studies provides the ability to gain insights into how pathogens such as *Salmonella* respond to feed additives in the presence of indigenous cecal microbiota. Importantly, evaluating multidrug-resistant (MDR) isolates within this *vitro* model offers critical insights into bacterial fitness under simulated cecal conditions. Using an *in vitro* poultry cecal model, we assessed the effect of SCFP on the reduction of six different MDR *Salmonella* serovars associated with poultry production (*S*. Typhimurium ATCC 14028, *S*. Enteritidis, *S*. Infantis, *S*. Heidelberg, *S*. Typhimurium DT104, and *S*. Reading) and the change in cecal microbial diversity and taxonomic compositional profiles.

## Materials and methods

### Poultry management and ceca collection

Ceca were aseptically collected from six 42-day-old broilers that were euthanized using CO₂. The broilers were fed a non-SCFP control diet formulated according to industry standards. Samples were obtained from the Cargill Innovation Center in Elk River, MN. The ceca were shipped overnight under anaerobic conditions using anaerobic sachets (Mitsubishi™ AnaeroPack-Anaero Gas Generator, ThermoScientific™, Waltham, MA, USA) to the Animal Science Building at the University of Wisconsin – Madison (Madison, WI, USA) where they were transported to an anaerobic chamber (90% N_2_, 5% CO_2_, and 5% H_2_) for processing (Coy Laboratory Products, Grass Lake, MI, USA).

### Media preparation

Anaerobic Dilution Solution (ADS; 0.45 g/L K_2_HPO_4_, 0.45 g/L KH_2_PO_4_, 0.45 g/L (NH_4_)SO_4_, 0.9 g/L NaCl, 0.1875 g/L MgSO_4_-7H_2_O, 0.12 g/L CaCl_2_-2H_2_O, 1 mL/L 0.1% resazurin, 0.05% cysteine-HCl and 0.4% sodium carbonate) was used as a general media [24,20]. Autoclaved ADS was adjusted to room temperature in an anaerobic chamber with the same atmosphere described above for 24 h. Resazurin in the ADS aided as a visual indicator that the media did not contain oxygen.

### Bacterial cultures

The *Salmonella enterica* serovars were sourced from the Cargill Innovation Center (Elk River, MN, USA). Six serovars were utilized in the current study: *S*. Typhimurium ATCC 14028, *S*. Enteritidis, *S*. Infantis, *S*. Heidelberg, *S*. Typhimurium DT104, and *S*. Reading. The serovars were grown on XLD agar supplemented with 30 μg/mL of novobiocin (NB) for 24 h at 37°C. The day before the inoculation of cecal contents, one colony of each serovar was transferred to 40 mL of Tryptic Soy Broth (Ward’s Science, Rochester, NY, USA) supplemented with 30 μg/mL of NB and incubated for 12 h at 37°C in a shaken incubator. On the day of the experiment, the cultures were centrifuged for 5 min at 18,0000 x g, and the pellets were subsequently resuspended in 20 mL ADS and vortexed. The antibiotic profiles of utilized MDR *Salmonella* serovars are described in Table 1.

**Table 1:** Multiple drug-resistant *Salmonella* serovars used in the current study with previously described antibiotic resistance to several antibiotics.

*Salmonella* serovar	Observed Antibiotic Resistance*	Reference
Typhimurium ATCC 14028	NA, AMP, STR, TET, Sulfa drugs	Narimisa et al., 2024; Alenazy, 2022 [24,25]
Enteritidis	AMP, CHL, STR, CEP, TET, KAN	Rakov and Kuznetsova, 2021 [26]
Infantis	TET, AMP, CEP, TMP-Sulfa, CHL, STR, Sulfa drugs	Alvarez et al., 2023 [27]
Heidelberg	AMC, AMP, CFX, CRO, STR, TET, AMC, CEF	Sielski Galvao Soares et al., 2023 [28]
Typhimurium DT104	AMP, CHL, STR, TET, Sulfa drugs	Meunier et al., 2002 [29]
Reading	NA, CIP	Sodagari et al., 2023 [30]

*Abbreviations to the antibiotics described in the table are as follows: nalidixic acid (NA), ampicillin (AMP), chloramphenicol (CHL), streptomycin (STR), sulfonamides (Sulfa drugs), tetracycline (TET), amoxicillin-clavulanic acid (AMC), cefoxitin (CFX), ceftriaxone (CRO), kanamycin (KAN), gentamicin (GEN), cefotaxime (CTX), cephalosporins (CEP), ceftiofur (CEF), ciprofloxacin (CIP).

### Anaerobic *in vitro* cecal assay

**Fig 1:**
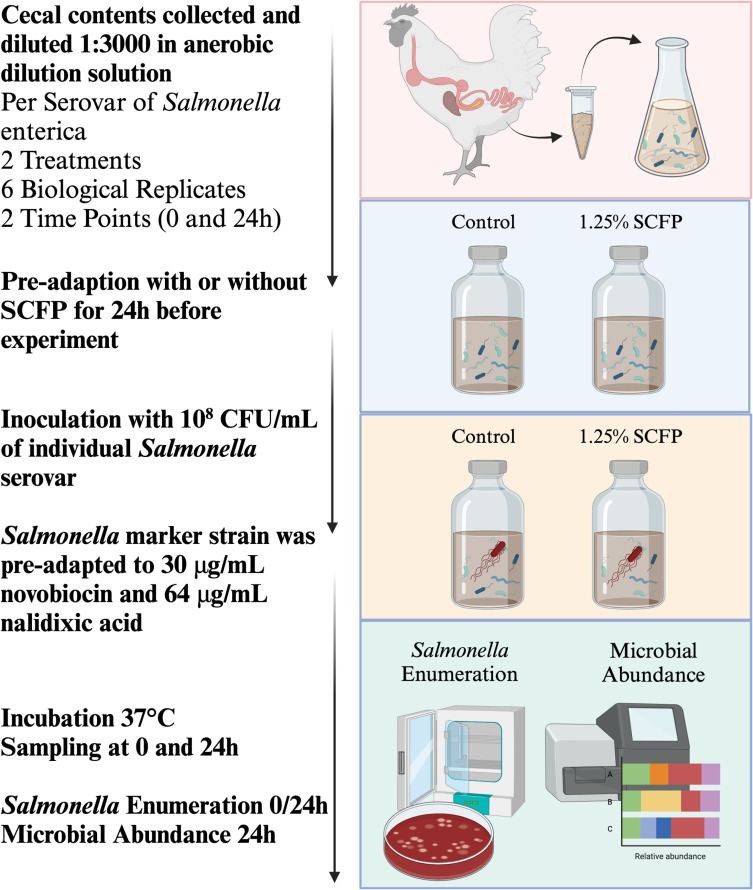
Overview of the experimental design in the current study. Created in BioRender, BioRender.com/p73k053.

A portion of cecal contents was excised at the ileocecal junction aseptically within the chamber, weighted, and diluted 1:3000 in ADS. Briefly, 0.1 g of cecal content was added to 900 μL ADS and vortexed. The resulting 1 mL of cecal suspension was added to 299 mL ADS for each cecum. A total of 20 mL of diluted cecal contents was transferred to each serum bottle containing appropriate treatments: 1) Control: 0.2 g of crushed poultry feed and 2) SCFP: 0.25 g of feed +  1.25% inclusion of Original XPC® (Diamond V, Cedar Rapids, IA, USA). The serum bottles were then covered with sterile rubber stoppers, removed from the anaerobic chamber, and incubated at 37°C for 24 h at 150 revolutions per minute (rpm) as a pre-adaptions step to acclimate the indigenous microbiota of the poultry ceca to the new environment as described by Donalson et al. (2007), Rubinelli et al. (2016), and Feye et al. (2020) [20,31,32]. The next day, the serum bottles were returned to the anaerobic chamber. Each bottle was then inoculated with 200 μL of an appropriate NB-resistant *Salmonella* serovar suspension (30 μg/mL), delivering a final concentration of 10⁶ colony-forming units (CFU)/mL. Duplicates of the samples were collected at 0 and 24 h for enumeration and microbiome analyses. The samples attained for the microbiome were flash-frozen in liquid nitrogen and stored at -80°C until the day of the processing. An overview of the process is described in Fig 1.

### Enumeration of *Salmonella* serovars and statistical analysis

The samples from 0 and 24 h (N =  144) were enumerated via spread plating on XLD agar supplemented with 30 μg/mL NB. The original samples were diluted to 10^-7^ in PBS, and 0.1 mL from the appropriate desired dilution series were spread-plated in duplicates on XLD with 30 μg/mL NB. The plates were incubated at 37°C for 24 h. The dilutions with countable colonies between 1 and 250 were calculated. The counts were log_10_ transformed before analyzing the data. A linear mixed effect (LME) model was utilized to assess the significant effect of treatment and time for each *Salmonella* serovar using the *lme4* package in RStudio Version 1.3. The significance was assumed at P <  0.05. The figures were created using Excel and BioRender.

### Microbiota sequencing, bioinformatics, and statistical analysis

DNA was from the aseptically collected aliquots of the anaerobic cultures taken at 24 h (N =  72) using Qiagen Qiamp Blood and Tissue Kit (Qiagen, Hilden, Germany). The DNA purity was measured via nanodrop and diluted to 10 ng/ µ L in AE buffer. The paired-end sequencing libraries were amplified, targeting the V4 region of the 16S rRNA gene with PCR primers containing the linker and adapter sequences [33]. DNA amplification was verified using gel electrophoresis. Normalization was performed on PCR products using the SequalPrep™ Normalization Kit (Life Technologies), ensuring equimolar concentrations of all libraries prior to pooling. Five µ L of each normalized sample was pooled to create a plate library. The libraries were evaluated for qualitative and quantitative homogeneity using a KAPA Library Quantification Kit (Kapa Biosystems, Inc., Wilmington, MA) to confirm uniform representation across samples. The resulting library was diluted to 20 nM and combined with HT1 Buffer, a PhiX control v3 (20 nM), and 0.2 N fresh NaOH to produce a final concentration of 6 pM. The subsequent sample was combined with PhiX control v3 (10%, v/v), and 600 µ L were loaded onto a MiSeq v2 (2 x 250 cycles) reagent cartridge (Illumina, San Diego, CA, United States).

Data sequences were uploaded onto the BaseSpace Website (Illumina, San Diego, CA, United States), where sequence run quality and run completion were assessed. Demultiplexed data were downloaded locally and uploaded into QIIME2-2021.11 via the Casava1.8 paired-end pipeline [34]. Data were visualized and trimmed in DADA2 using the chimera consensus pipeline [35]. Alpha and beta diversity were processed via the QIIME phylogeny align-to-tree-mafft-fasttree methodology and then examined for all available metrics of alpha and beta diversity via QIIME diversity core-metrics-phylogenetic with a sampling depth of 16460, determined based on rarefaction curves to ensure sufficient sequencing depth while maximizing sample retention (56% of features, 93% of samples) [36]. The taxonomic assignment of operational taxonomic units was conducted using classify-sklearn provided by the QIIME2-2021.11 SILVA database with a confidence limit of 97% [37,38]. Alpha diversity was evaluated for richness with the Shannon Diversity Index and evenness via Pielous’s Evenness [39]. The alpha diversity analytics involved the Kruskal-Wallis tests for pairwise differences within the variables and analysis of variance (ANOVA) to evaluate the main effect of variables [40]. The beta diversity metrics were tested with quantitative indicators, such as the Weighted Unifrac matrix and Bray-Curtis [41], using the Analysis of Similarity (ANOSIM) function, which considers the mean variation of the population and dispersion [42]. To account for potential dispersion effects in beta diversity comparisons, additional tests such as PERMANOVA were performed. The differential abundance was identified via ANCOM analysis [43]. To control for false discovery, Q-values were calculated using the Benjamini-Hochberg method, adjusting P-values for multiple comparisons with a strict FDR threshold (Q <  0.05) within the QIIME2 platform [44]. Microbiota main effects were considered significant if the main effect had P <  0.05 and the pairwise effect had Q <  0.05. All statistical analyses were conducted using QIIME2-2021.11, ensuring reproducibility within a standardized pipeline. Spearman’s correlation analysis on identified microbial taxa was performed in Excel. The sequences were uploaded to NCBI with accession number PRJNA1170279. The detailed report can be found within the metadata file that was uploaded to NCBI.

## Results

### Effect of SCFP on the reduction of MDR *Salmonella* serovars

Using an *in vitro* poultry cecal model, we assessed the effect of SCFP on the reduction of six different MDR *Salmonella* serovars that have previously been associated with poultry production (*S*. Typhimurium ATCC 14028, *S*. Enteritidis, *S*. Infantis, *S*. Heidelberg, *S*. Typhimurium DT104, and *S*. Reading) and the composition and diversity of the cecal microbiota. The cecal samples were pre-adapted to SCFP for 24 h, inoculated with the respective *Salmonella* serovar, and incubated at 37°C anaerobically for 24 hours.

**Fig 2:**
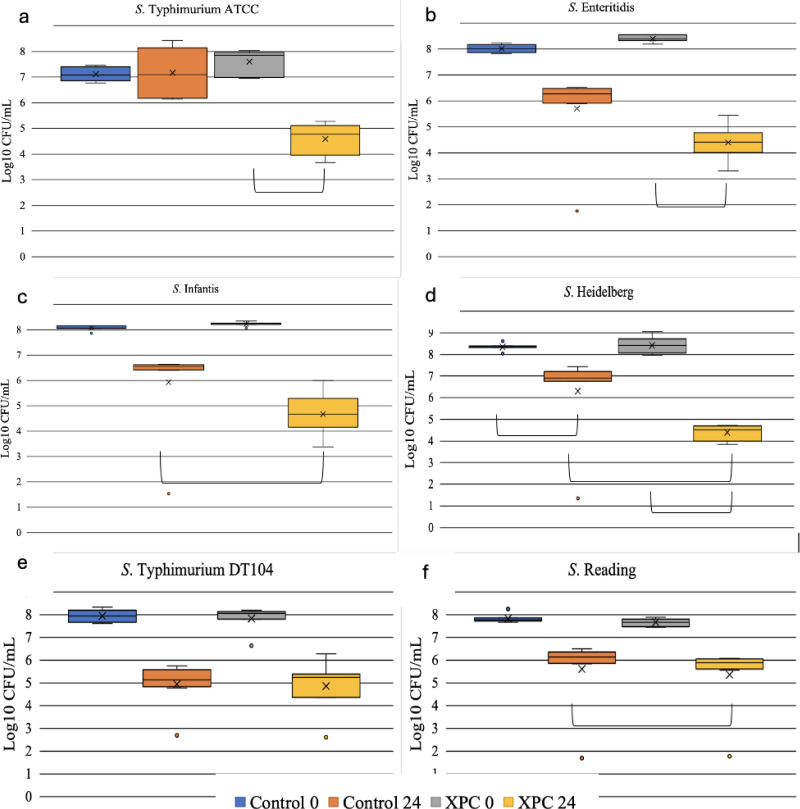
Effect of 1.25% of SCFP on MDR *Salmonella* serovars reduction at 24h. The serovars included *S*. Typhimurium ATCC#14028 (a), *S*. Enteritidis (b), *S*. Infantis (c), *S*. Heidelberg (d), *S*. Typhimurium DT104 (e), and *S*. Reading (f). The Y-axis represents the colony-forming units (CFU) per mL for each corresponding *Salmonella* serovar. Brackets indicate significant differences between treatments. Significant differences were determined using a linear mixed-effects model, with significance set at P <  0.05.

Treatment significantly affected the reduction of *S*. Typhimurium ATCC 14028, *S*. Enteritidis, *S*. Infantis, and *S*. Heidelberg (LME, P <  0.0001, S1 Table). In contrast, time significantly impacted the reduction of *S*. Typhimurium DT104 rather than treatment (P <  0.001). When comparing the reduction of *Salmonella* serovars at 24 hours based on the addition of SCFP to the control, *S*. Typhimurium ATCC 14028 was significantly reduced by 2.6 log_10_ CFU/mL, *S*. Enteritidis 1.8 log_10_ CFU/mL, *S*. Infantis 1.7 log_10_ CFU/mL, and *S*. Heidelberg 2.4 log_10_ CFU/mL (Fig 2a–2d). Additionally, *S*. Reading exhibited a significant reduction with the addition of SCFP (P =  0.03) at 24 hours compared to the control (0.3 log_10_ CFU/mL). The decrease was even more pronounced compared to the *Salmonella* counts in SCFP groups at 24 hours compared to the 0-hour time point. *S*. Typhimurium ATCC 14028 was reduced 3 log_10_ CFU/mL, *S*. Enteritidis 3.9 log_10_ CFU/mL, *S*. Infantis 3.4 log10 CFU/mL, *S.* Heidelberg 3.8 log_10_ CFU/mL, and *S*. Reading 1.8 log_10_ CFU/mL (Fig 2a–2d). Although various serovars exhibited somewhat different responses, SCFP effectively reduced multiple MDR serovars of *Salmonella*.

### Effect of SCFP on cecal microbiota composition in an *in vitro* poultry cecal model

**Fig 3:**
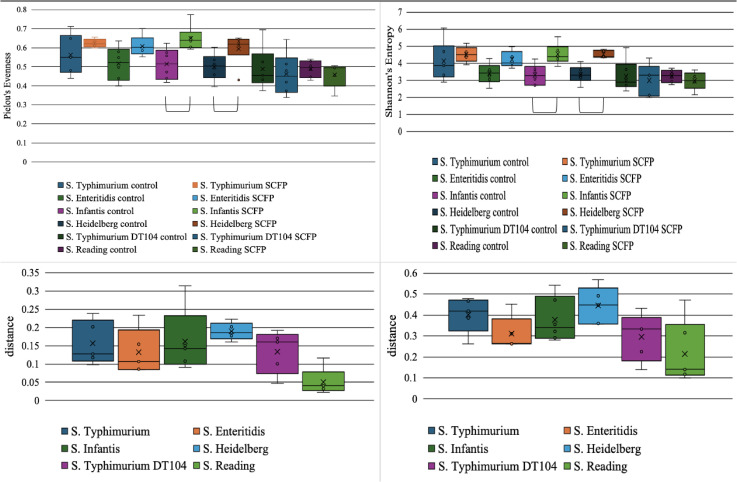
Effect of 1.25% SCFP on poultry cecal microbial composition inoculated with six multidrug-resistant (*MDR*) *Salmonella* serovars compared to a no-treatment control at 24 hours. The tested serovars included *S.* Typhimurium ATCC#14028, *S.* Enteritidis, *S.* Infantis, *S.* Heidelberg, *S.* Typhimurium DT104, and *S.* Reading. The top panels (a, b) illustrate microbial evenness (Pielou’s Evenness, *a*) and richness (Shannon’s Entropy, *b*) for each *Salmonella* serovar. The bottom panels (c, d) depict changes in diversity metrics when SCFP was added compared to the control. The left panel (*c*) represents Weighted UniFrac distance, which measures shifts in phylogenetic diversity, while the right panel (*d*) displays Bray-Curtis dissimilarity, assessing changes in microbial abundance. The X-axis in (*c*) and (*d*) corresponds to each *Salmonella* serovar, with bars indicating the magnitude of change in the respective metrics following SCFP supplementation. Both phylogenetic diversity and microbial abundance increased in samples where *Salmonella* reduction was significant with SCFP treatment. Brackets indicate significant differences between treatments (P <  0.05, Q <  0.05).

To discern the main effect of treatment on microbial compositions, we employed a one-way ANOVA approach related to evenness and richness parameters within the samples at 24 h. Post hoc pairwise comparisons were performed using the Tukey’s Honest Significant Difference (HSD) test to account for multiple testing corrections where applicable. These findings revealed a significant effect of treatment on cecal microbial evenness and richness compared to the control (Pielou’s Evenness, Shannon’s Entropy, P <  0.05). Interestingly, this change was only associated with the serovars where *Salmonella* counts were significantly reduced by at least 2 logs, such as *S*. Typhimurium ATCC, *S*. Enteritidis, *S.* Infantis, and *S*. Heidelberg. Compared to *S*. Typhimurium DT104 and *S*. Reading, the mean in evenness and richness was decreased with the addition of SCFP (Fig 3a and 3b). However, a significant increase in microbial diversity was only observed for *S.* Infantis and *S*. Heidelberg in the SCFP group compared to the control at 24h (P <  0.05, Fig 3). Additionally, ANOSIM was utilized to detect the main effect of treatment on phylogenetic composition and microbial abundance among the samples. These results indicated that phylogenetic composition and microbial abundance were significantly affected by SCFP (Weighted Unifrac, Bray-Curtis, P <  0.05). Phylogenetic diversity and microbial abundance increased in samples with 2-log *Salmonella* reduction (Fig 3c and 3d). However, a significant phylogenetic composition and abundance change was only observed in samples inoculated with *S.* Infantis and *S*. Heidelberg (P <  0.05, Fig 3c).

**Fig 4:**
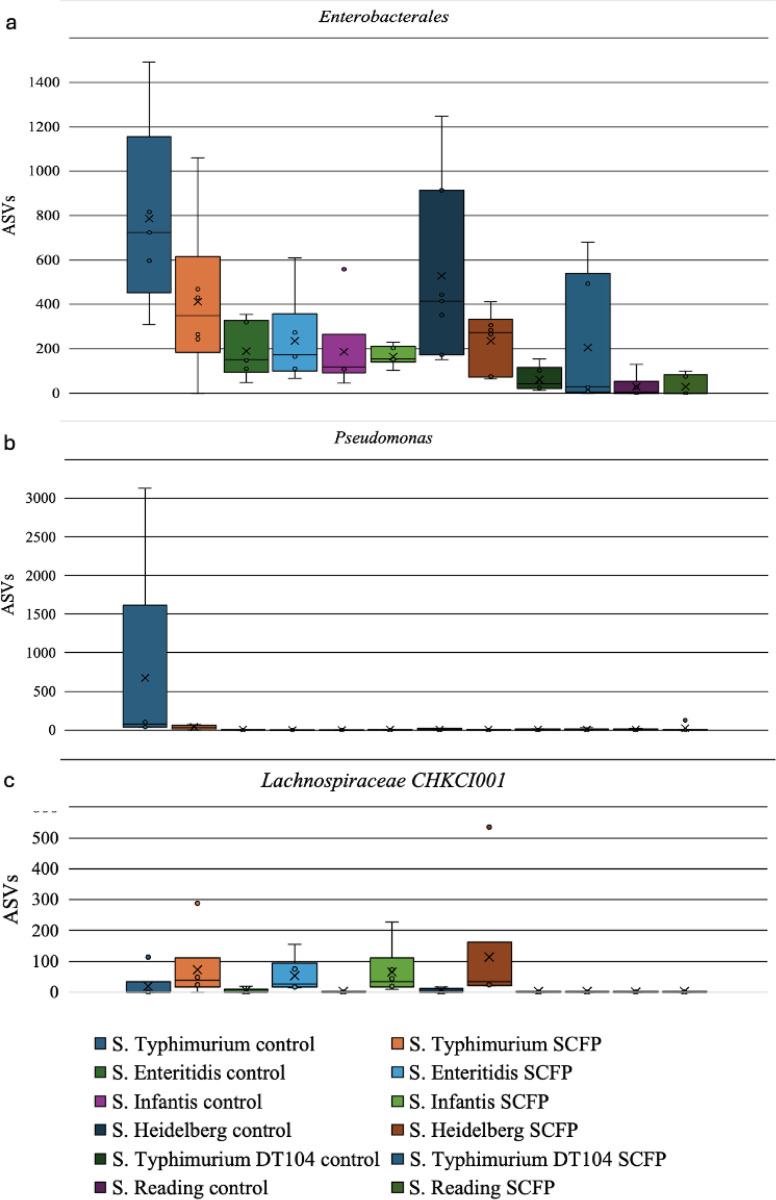
Microbial differential abundance at 24 hours post-treatment was assessed using ANCOM (*P* <  0.05). The tested serovars included *Salmonella* Typhimurium ATCC#14028, *S.* Enteritidis, *S.* Infantis, *S.* Heidelberg, *S.* Typhimurium DT104, and *S.* Reading. (a) The abundance of amplicon sequencing variants (ASVs) within *Enterobacterales* significantly decreased in the 1.25% SCFP treatment compared to the control at 24h, particularly in samples where *Salmonella* reduction was significant. (b) A *Pseudomonas* genus associated with *S.* Typhimurium ATCC#14028 was reduced in the SCFP treatment compared to the control. (c) *Lachnospiraceae* CHKC1001 abundance increased in the SCFP group compared to the control at 24h, specifically in serovars where *Salmonella* reduction was significant.

Out of the 212 identified taxa, the order *Enterobacterales* and the genera *Pseudomonas* and *Lachnospiraceae* CHKI001 were significantly differentiated in abundance based on SCFP (ANCOM, P <  0.05; S2 Table). The order *Enterobacterales* was decreased in SCFP-treated samples where *Salmonella* counts were significantly reduced compared to the control (Fig 4a). *Enterobacterales* were increased in control groups. The greatest reduction of *Enterobacterales* was observed in samples inoculated with *S*. Typhimurium ATCC 14028 and *S.* Heidelberg (Fig 4a). The genus *Pseudomonas* was notably increased in abundance in the control group in *S*. Typhimurium 14028 and significantly reduced with SCFP treatment (Fig 4b). Conversely, the genus *Lachnospiraceae* CHKI001 was abundant in SCFP samples where *Salmonella* counts were significantly reduced (Fig 4c). A Spearman’s correlation analysis revealed a very slight inverse relationship between *Enterobacterales* and *Lachnospiraceae* CHKI001 abundance in the samples where *Salmonella* was significantly reduced over 2 logs (Spearman’s correlation coefficient: -0.017). Overall, SCFP treatment improved the cecal system’s microbial diversity while effectively reducing *Salmonella*’s abundance.

## Discussion

### *Salmonella* reduction with the addition of SCFP

In our poultry cecal model, SCFP significantly reduced five out of six MDR *Salmonella* serovars, with reductions varying by strain. While most serovars exhibited an average decrease of 2 log₁₀ CFU/mL, S. Typhimurium DT104 showed no significant reduction. The emergence of MDR pathogenic variants of *S*. Typhimurium is an increasing concern due to their distinct host ranges, niche adaptations, levels of virulence, and risks to food safety [45]. One of the most common *Salmonella* phage types, *S*. Typhimurium definitive type (DT) 104, emerged as an antibiotic-resistant strain associated with human and animal health [46]. While the greatest reduction among serovars was achieved in *S*. Typhimurium inoculated cecal samples, the DT104 strain was not similarly reduced (Fig 2e). This finding suggests that *S*. Typhimurium serovars may have different metabolic interactions with the local microbiota and occupy distinct ecological niches. The genome of ATCC 14028 is typically stable and does not carry the MDR genes found in DT104. DT104 is a phage type of *S*. Typhimurium known for its multidrug resistance [47–49]. While DT104 shares many metabolic pathways with ATCC 14028, the presence of antibiotic-resistance genes may confer metabolic differences.

In the current study, *S*. Reading-inoculated cecal contents exhibited the least significant reduction of *Salmonella* (Fig 2f). *S*. Reading is more commonly associated with turkeys [50]. Feye et al. (2016) demonstrated a 5-log CFU/mL reduction of *S*. Reading with SCFP inclusion using a turkey *in vitro* model [12]. This niche preference may partially explain the minimal response to SCFP in the poultry cecal environment. Since *S*. Typhimurium, *S*. Enteritidis, *S*. Heidelberg, and *S*. Infantis are generally associated with chickens [51,52] and showed significant reductions in a chicken cecal *in vitro* model, indicating that serovar response may be host niche specific. This host distinction should be considered in challenge studies focusing on *Salmonella* intervention strategies.

### Microbiome response to MDR inoculated serovars with the addition of SCFP

Increased microbial abundance in *Enterobacterales* is a common indicator of dysbiosis [53]. *Salmonella* can disrupt the bacterial population hierarchy and become a dominant species [54,55]. Consequently, an increased diversity and abundance of local bacteria may impede the proliferation of *Salmonella*. The findings of the current study appear to support this hypothesis. We assessed the microbial composition and diversity by adding SCFP at 24 hours. Interestingly, microbial evenness, richness, abundance, and phylogenetic diversity increased in samples with SCFP and were associated with the four serovars where *Salmonella* reduction was over 2 logs (Fig 3c and 3d). In contrast, in samples where *Salmonella* was minimally reduced (*S*. Reading and *S*. Typhimurium DT104), microbial abundance, diversity, and overall composition were not impacted by SCFP. *S.* Reading’s niche specificity to the turkey cecal environment may explain the lack of change in microbial composition and diversity observed in the poultry cecal microbiota when inoculated with *S.* Reading in the current study. The minimal impact on the cecal microbial composition at 24 hours was accompanied by a minimal reduction in *S.* Reading counts based on the addition of SCFP. The effect of SCFP on microbial composition may affect the proliferation of pathogens such as *Salmonella* within the niche-specific environment. This observation is supported by differential abundance analysis, which indicated that the order *Enterobacterales* was increased in the control samples compared to the samples administered SCFP at 24 hours, where *Salmonella* abundance was significantly reduced over 2 logs (Fig 4a). Additionally, a genus of *Pseudomonas*, particularly associated with the *S*. Typhimurium ATCC strain, resulted in an increased abundance in control samples compared to SCFP (Fig 4b). This finding suggests that the impact of SCFP may extend beyond *Salmonella*, affecting other Gram-negative pathogens associated with the *Enterobacteriaceae* family. Supporting the hypothesis that increased local microbial diversity can impede the proliferation of pathogens [56]. While the current *in vitro* model evaluates only the impact of treatment on the microbiome, it does not consider the previously reported effects of SCFP on immune system modulation, the GIT epithelium, and tight junctions, which may enhance the overall effect.

Furthermore, the microbial composition response to SCFP was serovar-specific, with significant changes and increased evenness and richness observed in samples inoculated with *S*. Infantis and *S*. Heidelberg (Fig 3), suggesting a niche-specific response of *Salmonella* serovars within various GIT environments. For example, *S*. Enteritidis typically colonizes the upper GIT and invades the reproductive tract, leading to egg contamination [57–59]. While *S*. Typhimurium is associated with various organs, *S*. Infantis and *S*. Heidelberg are commonly found in the colon and cecum of poultry [60–63]. The composition of the GIT microbiota can influence the ability of *Salmonella* to proliferate in different compartments of the GIT, thus affecting the variations of SCFP on microbial composition in our cecal *in vitro* model. Moreover, a genus of *Lachnospiraceae* CHKCI001 was increased in abundance in samples administered SCFP compared to control in the samples inoculated with serovars where *Salmonella* abundance was significantly reduced over 2 logs. Although previous research showed that *Enterobacterales* is inversely proportional to *Lachnospiraceae* [64,65], the findings of the current study indicated a very slight inverse relationship between the two taxa when considering the serovars where *Salmonella* was significantly reduced by 2 logs with SCFP compared to control at 24 h. This finding suggests that the relationship between *Enterobacterales* and *Lachnospiraceae* CHKCI001 might be mediated by interactions with other microbial taxa, further supporting the hypothesis that increased indigenous microbial diversity may affect the abundance of pathogens such as *Salmonella.* Thus, the effect of SCFP on the indigenous microbiota can indirectly affect the abundance of *Salmonella*.

## Conclusions

The current study assessed the impact of the postbiotic feed additive SCFP on reducing MDR *Salmonella* serovars using an *in vitro* poultry cecal model. The results demonstrated a significant reduction in *Salmonella* populations for five out of the six serovars examined, specifically *S*. Typhimurium ATCC 14028, *S*. Enteritidis, *S*. Infantis, and *S*. Heidelberg, with an average reduction of 2 log_10_ CFU/mL after 24 hours of incubation with SCFP and *S*. Reading with 0.3 log_10_ CFU/mL compared to control at 24 h. In contrast, *S*. Typhimurium DT104 did not result in a significant reduction based on treatment. The differential responses among serovars suggest that the metabolic interactions with local microbiota and niche adaptations play a crucial role in the efficacy of SCFP.

The study also highlighted that SCFP administration increased microbial diversity and abundance in samples with significant *Salmonella* reduction. This finding suggests that enhancing indigenous microbial diversity can impede pathogen proliferation. The serovar-specific microbial compositional responses further emphasize the importance of considering niche-specific interactions in designing intervention strategies. SCFP has long been recognized for its ability to reduce *Salmonella*. Our findings further support this knowledge by confirming its effectiveness against *Salmonella* strains that harbor multidrug resistance. Although there were detectable serovar-specific influences, the data demonstrate the broad positive impact of SCFP as a preharvest intervention against *Salmonella* and other potential pathogens.

## Supporting information

S1 Table Significant treatment effects and pairwise differences (via Linear Mixed Effect (LME) model) on multiple drug-resistant Salmonella serovars used in the current study. (DOCX)

S2 Table Taxa associated with each serovar (Typhimurium ATCC14028 (S1), Enteritidis (S2), Infantis (S3), Heidelberg (S4), Typhimurium DT104 (S5), Reading (S6)) based on treatment that included control (C) or addition of XPC (SCFP). Averages of six replicates per treatment per serovar. (DOCX)
