# Supplementary material for: Potential of saccharomyces cerevisiae fermentation-derived postbiotic technology in mitigating multiple drug-resistant Salmonella enterica serovars in an in vitro broiler cecal model
Source: PLoS One. 2025 Apr 3;20(4):e0320977. doi: 10.1371/journal.pone.0320977 (PMC11967930; doi:10.1371/journal.pone.0320977)
Supplement: S1 Table — (DOCX) [file pone.0320977.s001.docx]

|  |  |  | **Estimate** | **SE**^1^ | **df**^2^ | **t-value**^3^ | **p-value**^4^ |
| --- | --- | --- | --- | --- | --- | --- | --- |
|  |  | Intercept | 8.0178 | 1.3404 | 20 | 5.982 | 7.54e-6 |
|  |  | Treatment XPC | 0.3829 | 0.2301 | 20 | 1.664 | 0.112 |
|  | Fixed Effects | Time 24h | -1.7514 | 1.8956 | 20 | -0.924 | 0.367 |
|  |  | Treatment XPC: Time 24h | -2.1802 | 0.3253 | 20 | -6.701 | 1.60e-6 |
| **Enteritidis** |  | Control – XPC at 0h | -0.383 | 0.23 | 20 | -1.664 | 0.116 |
|  |  | Control – XPC at 24h | 1.797 | 0.23 | 20 | 7.813 | < 0.0001 |
|  | Pairwise | Control: Time0 – Time24 | 1.75 | 1.75 | 92205 | 0.924 | 0.3555 |
|  |  | XPC: Time0 – Time24 | 3.93 | 3.93 | 92205 | 2.074 | 0.0381 |
|  |  | Intercept | 8.0748 | 1.6739 | 20 | 4.824 | 0.000103 |
|  |  | Treatment XPC | 0.1571 | 0.2185 | 20 | 0.719 | 0.480462 |
|  | Fixed Effects | Time 24h | -1.5209 | 2.3673 | 20 | -0.642 | 0.527854 |
|  |  | Treatment XPC: Time 24h | -1.8551 | 0.309 | 20 | -6.004 | 7.18e-6 |
| **Infantis** |  | Control – XPC at 0h | -0.157 | 0.218 | 20 | -0.719 | 0.4805 |
|  |  | Control – XPC at 24h | 1.698 | 0.218 | 20 | 7.772 | < 0.0001 |
|  | Pairwise | Control: Time0 – Time24 | 1.52 | 2.37 | 275688 | 0.642 | 0.5206 |
|  |  | XPC: Time0 – Time24 | 3.38 | 2.37 | 275688 | 1.426 | 0.1538 |
|  |  | Intercept | 8.35337 | 0.43885 | 16 | 19.035 | 2.05e-12 |
|  |  | Treatment XPC | 0.07601 | 0.17319 | 16 | 0.439 | 0.6666 |
|  | Fixed Effects | Time 24h | -1.33703 | 0.62063 | 16 | -2.154 | 0.0468 |
|  |  | Treatment XPC: Time 24h | -2.50076 | 0.29997 | 16 | -8.337 | 3.24e-7 |
| **Heidelberg** |  | Control – XPC at 0h | -0.076 | 0.173 | 16 | -0.439 | 0.6666 |
|  |  | Control – XPC at 24h | 2.425 | 0.245 | 16 | 9.9 | < 0.0001 |
|  | Pairwise | Control: Time0 – Time24 | 1.34 | 0.621 | 2638 | 2.154 | 0.0313 |
|  |  | XPC: Time0 – Time24 | 3.84 | 0.644 | 766 | 5.956 | < 0.0001 |
|  |  | Intercept | 7.10808 | 0.49832 | 20 | 14.264 | 6.06e-12 |
|  |  | Treatment XPC | 0.49561 | 0.36414 | 20 | 1.361 | 0.189 |
|  | Fixed Effects | Time 24h | 0.05831 | 0.70474 | 20 | 0.083 | 0.935 |
|  |  | Treatment XPC: Time 24h | -3.0655 | 0.51498 | 20 | -5.953 | 8.0e-6 |
| **Typhimurium ATCC** |  | Control – XPC at 0h | -0.496 | 0.364 | 20 | -1.361 | 0.1886 |
|  |  | Control – XPC at 24h | 2.57 | 0.364 | 20 | 7.057 | < 0.0001 |
|  | Pairwise | Control: Time0 – Time24 | -0.0583 | 0.705 | 281 | -0.083 | 0.9341 |
|  |  | XPC: Time0 – Time24 | 3.0072 | 0.705 | 281 | 4.267 | < 0.0001 |
|  |  | Intercept | 7.9482 | 0.4985 | 19 | 15.943 | 1.87e-12 |
|  |  | Treatment XPC | -0.1204 | 0.2964 | 19 | -0.406 | 0.68917 |
|  | Fixed Effects | Time 24h | -2.6999 | 0.705 | 19 | -3.829 | 0.00113 |
|  |  | Treatment XPC: Time 24h | 0.1082 | 0.4296 | 19 | 0.252 | 0.8038 |
| **Typhimurium DT104** |  | Control – XPC at 0h | 0.1204 | 0.296 | 19 | 0.406 | 0.6892 |
|  |  | Control – XPC at 24h | 0.0122 | 0.311 | 19 | 0.039 | 0.9692 |
|  | Pairwise | Control: Time0 – Time24 | 2.7 | 0.705 | 608 | 3.839 | 0.0001 |
|  |  | XPC: Time0 – Time24 | 2.59 | 0.711 | 520 | 3.644 | 0.0003 |

^1^ Standard Deviation

^2^ Degrees of Freedom

^3^ T-value difference relative to variance

^4^ P-Value main effect differences
