# Supplementary material for: Potential of saccharomyces cerevisiae fermentation-derived postbiotic technology in mitigating multiple drug-resistant Salmonella enterica serovars in an in vitro broiler cecal model
Source: PLoS One. 2025 Apr 3;20(4):e0320977. doi: 10.1371/journal.pone.0320977 (PMC11967930; doi:10.1371/journal.pone.0320977)
Supplement: S2 Table — (DOCX) [file pone.0320977.s002.docx]

|  | **S1_C** | **S1_SCFP** | **S2_C** | **S2_SCFP** | **S3_C** | **S3_SCFP** | **S4_C** | **S4_SCFP** | **S5_C** | **S5_SCFP** | **S6_C** | **S6_SCFP** |
| --- | --- | --- | --- | --- | --- | --- | --- | --- | --- | --- | --- | --- |
| **Oscillospiracaeae** | 373.50 | 560.67 | 355.83 | 421.83 | 245.17 | 508.50 | 311.50 | 594.67 | 472.0 | 272.17 | 519.50 | 256.0 |
| **Incertae sedis** | 63.50 | 196.21 | 64.96 | 117.83 | 46.17 | 221.67 | 49.50 | 132.17 | 50.67 | 39.17 | 95.50 | 29.17 |
| **Clostridia** | 252.33 | 161.0 | 99.67 | 100.33 | 97.33 | 122.67 | 121.67 | 102.0 | 146.67 | 100.83 | 134.67 | 99.50 |
| **Ruminococcaceae** | 470.0 | 574.83 | 396.50 | 339.83 | 291.67 | 678.33 | 417.83 | 392.67 | 401.83 | 327.50 | 348.0 | 141.83 |
| **Lachnospiraceae** | 3225.50 | 4619.33 | 3442.85 | 3414.33 | 2389.33 | 4396.33 | 2831.33 | 3928.83 | 2840.17 | 1763.0 | 3381.17 | 2063.50 |
| **Clostridia UCG-014** | 102.50 | 131.17 | 26.83 | 33.33 | 19.0 | 52.33 | 39.50 | 43.83 | 43.0 | 62.33 | 36.0 | 46.17 |
| **Akkermansia** | 40.50 | 4.83 | 1.67 | 1.0 | 2.33 | 1.17 | 5.50 | 19.17 | 3.67 | 3.33 | 53.50 | 15.83 |
| **[Eubacterium] coprostanoligenes** | 2.33 | 16.5 | 1.33 | 13.33 | 0.83 | 12.83 | 0.83 | 21.83 | 1.50 | 2.33 | 20.67 | 1.33 |
| **CHKCI001** | 20.0 | 73.17 | 4.33 | 52.33 | 1.50 | 65.67 | 4.50 | 113.67 | 1.83 | 1.0 | 0.67 | 1.33 |
| **Oscillibacter** | 111.50 | 116.0 | 80.0 | 78.33 | 53.33 | 78.33 | 136.33 | 80.17 | 61.17 | 99.50 | 55.33 | 74.83 |
| **Oscillospirales** | 60.33 | 45.50 | 38.33 | 19.33 | 83.0 | 18.0 | 45.0 | 34.17 | 38.33 | 26.0 | 85.83 | 34.83 |
| **Bacteroides** | 12009.83 | 7512.50 | 15619.67 | 7571.67 | 13018.33 | 6740.0 | 15151.0 | 6804.0 | 13117.17 | 10464.5 | 15750.0 | 12530.33 |
| **Pseudomonas** | 566.67 | 31.33 | 1.17 | 0 | 0 | 1.83 | 6.0 | 1.67 | 3.0 | 7.67 | 6.83 | 21.0 |
| **Rhodospirillales uncultured** | 23.17 | 1.17 | 12.67 | 1.0 | 12.0 | 2.67 | 23.0 | 1.50 | 11.17 | 2.50 | 9.67 | 2.67 |
| **Clostridium_sensu_strico_1** | 162.17 | 1145.83 | 144.33 | 945.17 | 281.33 | 993.83 | 247.33 | 1216.67 | 108.0 | 26.50 | 362.83 | 132.0 |
| **Peptostreptococcaceae** | 193.33 | 20.17 | 10.33 | 0.67 | 13.0 | 4.50 | 14.0 | 4.50 | 16.83 | 23.83 | 27.83 | 22.83 |
| **Parasutterella** | 182.17 | 224.83 | 210.33 | 152.17 | 119.83 | 191.50 | 157.33 | 232.17 | 69.17 | 37.50 | 72.83 | 55.33 |
| **Lactobacilus** | 1304.83 | 1075.17 | 178.0 | 407.83 | 233.17 | 672.17 | 284.0 | 805.50 | 301.0 | 1023.67 | 379.83 | 132.0 |
| **Gordonibacter** | 1.50 | 26.17 | 2.67 | 21.83 | 1.50 | 22.83 | 5.50 | 19.67 | 5.0 | 1.67 | 6.67 | 1.83 |
| **Bifidobacterium** | 479.33 | 122.83 | 525.67 | 153.0 | 785.67 | 269.33 | 311.50 | 129.67 | 332.0 | 102.17 | 383.50 | 266.33 |
| **Erysipelatoclostridium** | 66.67 | 104.67 | 55.0 | 71.0 | 26.83 | 72.83 | 59.5 | 92.83 | 27.83 | 32.0 | 26.33 | 18.0 |
| **Corynebacteriaceae** | 27.50 | 29.33 | 12.67 | 7.33 | 16.0 | 10.0 | 16.83 | 4.33 | 4.50 | 29.83 | 10.17 | 30.0 |
| **Slackia** | 61.33 | 40.83 | 65.83 | 59.67 | 41.0 | 59.67 | 54.67 | 25.67 | 59.67 | 52.83 | 77.50 | 53.17 |
| **Enterobacterales** | 725.0 | 411.33 | 189.0 | 234.33 | 185.33 | 165.67 | 546.67 | 234.17 | 62.5 | 206.17 | 27.5 | 29.17 |
| **Frisingicoccus** | 31.17 | 5.83 | 46.50 | 12.67 | 40.83 | 5.33 | 63.67 | 6.0 | 28.17 | 24.50 | 96.83 | 38.50 |
| **Subdoligranulum** | 1200.83 | 1445.0 | 1157.17 | 922.5 | 1007.67 | 1380.83 | 973.33 | 1007.83 | 1265.33 | 921.33 | 1301.67 | 1014.33 |
| **Uncultured** | 24.0 | 20.33 | 13.0 | 10.67 | 7.17 | 11.50 | 8.17 | 13.0 | 4.33 | 8.50 | 1.33 | 1.83 |
| **GCA-900066575** | 12.33 | 41.83 | 3.83 | 10.67 | 3.50 | 33.50 | 4.50 | 25.83 | 7.67 | 5.67 | 5.83 | 3.17 |
| **Bacteria Firmicutes** | 27.33 | 29.33 | 13.0 | 22.83 | 8.0 | 40.33 | 12.67 | 25.50 | 16.50 | 9.5 | 16.17 | 20.5 |
| **Anaerovoracaceae** | 19.83 | 8.83 | 12.67 | 11.33 | 19.67 | 14.17 | 20.67 | 10.50 | 17.33 | 9.17 | 17.33 | 15.50 |
| **Bilophila** | 194.67 | 100.83 | 163.17 | 96.33 | 149.67 | 91.17 | 205.67 | 62.17 | 157.33 | 71.33 | 165.17 | 135.0 |
| **Bacilli** | 171.83 | 50.67 | 10.17 | 23.50 | 14.33 | 30.67 | 32.33 | 30.50 | 4.0 | 282.17 | 22.33 | 19.0 |
| **Eggerthellaceae** | 7.0 | 14.17 | 5.17 | 16.50 | 4.0 | 16.17 | 3.17 | 14.33 | 7.67 | 3.50 | 4.0 | 4.83 |
| **Butricioccus** | 158.33 | 285.0 | 224.0 | 249.67 | 133.0 | 272.50 | 144.67 | 238.67 | 141.83 | 80.67 | 125.0 | 72.17 |
| **Lachnospirales** | 124.83 | 116.67 | 81.17 | 83.33 | 61.50 | 127.83 | 116.17 | 101.50 | 112.67 | 44.0 | 97.17 | 77.17 |
| **Enterobacteriaceae** | 4267.17 | 2799.0 | 4406.17 | 2739.0 | 4177.0 | 2913.33 | 4709.67 | 2013.17 | 4769.50 | 3294.33 | 7025.0 | 5659.17 |
| **Butyricioccaceae** | 5.0 | 57.17 | 1.50 | 34.83 | 0 | 37.83 | 0 | 32.0 | 0 | 0 | 0 | 0 |
| **Christensenellaceae**  **R-7** | 16.33 | 28.33 | 8.83 | 38.83 | 6.83 | 24.33 | 6.83 | 26.17 | 18.17 | 6.67 | 9.33 | 2.17 |
| **Anarotruncus** | 18.17 | 15.17 | 14.33 | 16.17 | 14.50 | 14.67 | 14.33 | 15.0 | 16.83 | 8.0 | 19.17 | 9.17 |
| **Alistipes** | 186.83 | 80.67 | 115.17 | 67.33 | 107.33 | 64.50 | 90.50 | 60.50 | 96.17 | 77.67 | 152.83 | 72.83 |
| **Enterorhabdus** | 5.83 | 17.67 | 2.83 | 17.67 | 3.67 | 19.50 | 5.17 | 12.67 | 7.67 | 0.67 | 9.50 | 5.83 |
| **Desulfovibrionaceae** | 14.0 | 20.33 | 8.17 | 11.17 | 7.0 | 12.67 | 5.33 | 7.0 | 9.17 | 5.17 | 11.0 | 5.0 |
| **CHKCI002** | 140.0 | 80.50 | 140.83 | 92.50 | 86.33 | 85.0 | 136.33 | 72.5 | 129.33 | 68.67 | 140.50 | 97.33 |
| **Streptococcus** | 1601.67 | 2161.83 | 767.33 | 1493.33 | 761.67 | 2491.67 | 735.83 | 2097.67 | 683.67 | 574.33 | 795.0 | 304.67 |
| **Sellimonas** | 13.83 | 32.83 | 10.33 | 23.33 | 13.17 | 23.17 | 9.17 | 31.83 | 21.83 | 3.50 | 8.83 | 8.0 |
| **Anaerofilum** | 4.33 | 13.33 | 10.67 | 10.83 | 6.83 | 21.67 | 10.33 | 12.83 | 8.67 | 6.83 | 6.17 | 1.17 |
| **Anaerostipes** | 36.33 | 37.50 | 240.83 | 36.0 | 155.50 | 79.17 | 90.67 | 74.0 | 14.67 | 13.30 | 6.83 | 2.17 |
| **Merdibacter** | 14.33 | 8.50 | 13.50 | 5.0 | 7.0 | 7.50 | 11.83 | 6.33 | 6.67 | 2.67 | 5.17 | 0 |
| **[Ruminococcus] gauvreauii** | 22.83 | 28.5 | 17.33 | 16.83 | 12.50 | 29.50 | 16.67 | 21.83 | 10.50 | 7.17 | 9.50 | 3.50 |
| **Enterococcus** | 1852.83 | 4661.50 | 2354.0 | 2844.0 | 1332.5 | 3433.33 | 1666.5 | 3882.5 | 546.83 | 388.0 | 822.33 | 556.50 |
| **Pasteurellaceae** | 34.17 | 1.0 | 57.67 | 9.67 | 34.50 | 11.67 | 42.0 | 13.17 | 13.17 | 8.50 | 37.17 | 28.0 |
| **Other*** | 1567.83 | 304.50 | 122.50 | 381.33 | 62.50 | 111.0 | 94.17 | 355.0 | 101.67 | 55.0 | 349.0 | 142.50 |

* Summed averages per serovar of taxa with low detection values
